# Proteome Analysis of Seven *Treponema pallidum* subsp. *pallidum* Strains Grown *In Vitr* *o*

**DOI:** 10.1021/acs.jproteome.5c00624

**Published:** 2025-10-10

**Authors:** Juraj Bosák, Matěj Hrala, Klára Janečková, Kateřina Hanáková, Petra Pospíšilová, David Potěšil, Petr Andrla, Zbyněk Zdráhal, David Šmajs

**Affiliations:** 1 Department of Biology, Faculty of Medicine, 37748 Masaryk University, Brno 625 00, Czech Republic; 2 National Centre for Biomolecular Research, Central European Institute of Technology and Faculty of Science, 37748 Masaryk University, Brno 625 00, Czech Republic

**Keywords:** *Treponema pallidum*, *Treponema pallidum* subsp. *pallidum*, syphilis, *in vitro*, proteome

## Abstract

*Treponema pallidum* subsp. *pallidum* (*T. pallidum*), the fastidious causative agent of syphilis, has become more accessible for research with its recently developed *in vitro* cultivation method. In this work, the proteomes of seven *T. pallidum* strains (Nichols-like: DAL-1, Haiti B, and Madras; SS14-like: SS14, Mexico A, Philadelphia 1, and Grady), cultivated *in vitro*, were analyzed in biological triplicates by liquid chromatography–tandem mass spectrometry (LC–MS/MS). The MS/MS data were processed against their corresponding genomes using various annotation algorithms (DFAST, PGAP, Prodigal, Prokka, RAST, GeneMarkS, and manual GenBank annotation). Additionally, ORFfinder was used to predict all ORFs encoding polypeptides exceeding 50 amino acids. While the RAST algorithm predicted the highest number of genes per genome, GeneMarkS offered the best coverage of annotated genes (up to 88.9%). By combining annotations from seven *T. pallidum* strains, we identified 911 unique treponemal proteins (74.9% of 1216 predicted sequences). The confidence of protein identifications was high, with 85.5% identified by two or more peptides and 72.4% by three or more peptides. Overall, 51 proteins showed statistically significant quantitative differences in intensity across *T. pallidum* strains. Furthermore, our proteome analysis revealed detectable quantitative proteomic differences between strains in the Nichols-like and SS14-like groups.

## Graphical abstract

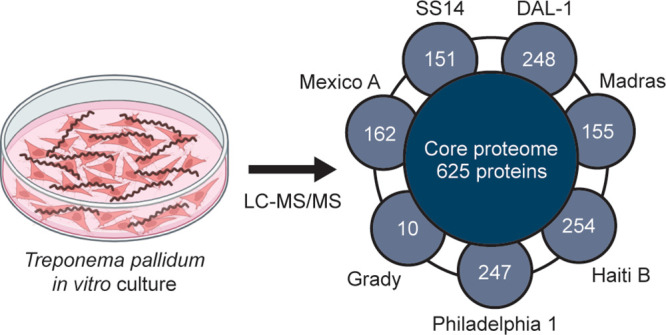

## Introduction

*Treponema pallidum* subsp. *pallidum* (*T. pallidum*, TPA) is the causative agent of syphilis, a sexually transmitted disease infecting humans worldwide and showing a rising trend in the recent decade, including Europe and North America.^1,2^

Historically, several attempts were made to analyze the *T. pallidum* proteome, mostly focusing on a single strain propagated in infected rabbit. In 2010, McGill et al.^3^ analyzed the proteome of the Nichols strain from infected rabbits. Using two-dimensional gel electrophoresis and matrix-assisted laser desorption ionization-time-of-flight (MALDI-TOF) analysis, they detected a total of 88 treponemal proteins in the analyzed spots. In 2016, Osbak et al.^4^ analyzed the proteome of strain DAL-1 propagated in rabbits by MALDI-TOF/TOF and electrospray ionization (ESI-LTQ-Orbitrap) tandem mass spectrometry. They found 557 unique treponemal proteins, including 114 annotated as hypothetical or uncharacterized proteins. Recently, the study by Houston et al.,^5^ which analyzed the proteome of Nichols strain propagated in rabbits by liquid chromatography-tandem mass spectrometry (LC–MS/MS), identified a total of 758 proteins, including 175 proteins classified as hypothetical or of unknown function. Later, Houston et al.^6^ analyzed and compared the proteome of the Nichols strain grown under both *in vivo* and *in vitro* conditions by LC–MS/MS and detected a total of 924 treponemal proteins, including 230 hypothetical proteins and proteins of unknown function, as well as 39 miniproteins (defined as proteins shorter than 150 amino acid residues) of unknown function. In the most recent study, Houston et al.^7^ analyzed the *in vitro* proteome of strain SS14 by LC–MS/MS and found similar protein profile when compared to the Nichols strain. However, only approximately two-thirds of predicted SS14 strain proteins were detected and quantitated. Combined results from all these studies revealed a 95% proteome coverage of *T. pallidum,* suggesting that a vast majority of predicted *T. pallidum* proteins are synthesized and detectable by the current proteomic tools.

The development of the continuous cultivation of *T. pallidum* using a coincubation with rabbit epithelial cells revolutionized the work with this fastidious pathogen.^8^ Despite being technically challenging, this *in vitro* cultivation system enables the simultaneous preparation of multiple treponemal strains with sufficient cell density, allowing for biological and technical replicates.

Genetic analyses of treponemal reference strains as well as human clinical isolates revealed two genetically distinct groups of *T. pallidum* strains, one related to the reference strain Nichols (Nichols-like strains) and the other to the reference strain SS14 (SS14-like strains).^9–13^ Based on the comparison of Nichols and SS14 genomes,^10^ both strains differ at more than 600 nucleotide positions and this difference opened the question of whether the Nichols-like and SS14-like strains differ also on the proteome level.

The aim of this study was to compare the proteomes of seven *T. pallidum* strains propagated *in vitro*, encompassing both Nichols-like and SS14-like groups. Using LC-MS/MS, we revealed quantitative proteome differences among individual strains and also between Nichols-like and SS14-like strains.

## Materials and Methods

### Source of *T. pallidum* Strains and Conditions for *In Vitro* Cultivation

The *T. pallidum* strains in this study (Table 1) were obtained from frozen samples (−80 °C, 15% glycerol) of infected rabbit testes within our laboratory stock.

**Table 1:** *T. pallidum* (TPA) Strains Analyzed in This Study^a^

TPA strain	place and year of isolation	source	TPA cluster^b^	GenBank accession number
Haiti B	Cotes-de-Fer, Haiti; 1951	Cox, D.L., CDC Atlanta, USA	Nichols-like	CP032623.1
Madras	Madras, India; 1954	Cox, D.L., CDC Atlanta, USA	Nichols-like	CP078121.1
DAL-1	Dallas, Texas, USA; 1991	Cox, D.L., CDC Atlanta, USA	Nichols-like	CP003115.1
Mexico A	unknown city, Mexico; 1953	Hawley K., UConn, Farmington, USA	SS14-like	CP003064.1
SS14	Atlanta, Georgia, USA; 1977	Hawley K., UConn, Farmington, USA	SS14-like	CP004011.1
Grady	Grady Hospital in Atlanta, USA; 1980s	Cox, D.L., CDC Atlanta, USA	SS14-like	CP035104.1
Philadelphia 1	Philadelphia, USA; 1988	Cox, D.L., CDC Atlanta, USA	SS14-like	CP035193.1

a Notes: CDC - Centers for Disease Control and Prevention; UConn - University of Connecticut School of Medicine.

b Based on Nechvátal et al.^9^

*T. pallidum* strains were continuously cultivated *in vitro* using a previously published cultivation system^8^ with few modifications.^14^ Briefly, treponemes were cultivated in a well (6-well plate, Cat. Number 92406, Techno Plastic Products) containing TpCM-2 medium (4 mL) and a monolayer of rabbit cells (Sf1Ep; 50,000 cells). At seven-day intervals, the treponemal cultures were detached (2 × 500 μL of Trypsin-EDTA, 37 °C, 5 min) and centrifuged for depletion of Sf1Ep cells (100 × g, 5 min). The supernatant containing treponemes (5 μL aliquot) was subjected immediately to dark-field microscopy (see below) and then inoculated (250–500 μL) to a freshly prepared well and cultivated at 34 °C in a low oxygen atmosphere (2.5%).

### Dark-Field Microscopy (DFM) for the Detection and Quantitation of Treponemes

In treponemal samples (5 μL), treponemes were counted at 400× magnification using a BX53 microscope (Olympus Corporation). Five individual fields were analyzed for each sample, and the average number of treponemes was calculated. Based on the size of the visual field and sample volume under the coverslip, one treponeme per field represented 340,000 treponemes per mL of culture.

### Sample Preparation of *T. pallidum* Strains for Proteome Analysis

The long-term *in vitro* cultures were used as the source of treponemes (see above). To minimize variability among treponemal cultures, each *T. pallidum* strain was transferred into a T75 flask (Cat. Number 90075, Techno Plastic Products). Here, treponemes (4 × 10^6^ treponemal cells) were cultivated with Sf1Ep cells (250,000 cells) in TpCM-2 medium (10 mL) in a low-oxygen atmosphere (2.5%). After 7 days of cultivation, treponemes were harvested (2 × 2 mL of Trypsin-EDTA, 37 °C, 5 min), centrifuged to deplete Sf1Ep cells (100 × g, 5 min), and the supernatant (3 mL) was inoculated into a freshly prepared cultivation in a T75 flask (Sf1Ep cells: 250,000; TpCM-2 medium: 10 mL), which was further cultivated for 7 days in a low-oxygen atmosphere (2.5%). Then, treponemal cultures were harvested for proteome analysis using 2 mL of dissociation medium without trypsin (34 °C, 2.5% O_2_ atmosphere, 30 min).^15^ After depletion of Sf1Ep cells (100 × g, 5 min), the treponemal suspension was collected (12 mL) and concentrated in PBS (10,000 × g for 5 min, repeated 5 times), resulting in a final volume of 200 μL. Treponemal numbers were quantified by dark-field microscopy (see above), and the sample was immediately frozen (−20 °C). The experiment was performed subsequently in three biological replicates for each *T. pallidum* culture.

### Liquid Chromatography-Tandem Mass Spectrometry (LC-MS/MS) Analyses

Harvested treponemal samples (100 μL; see above) were thawed, mixed with lysis buffer (200 μL; 4% SDS, 0.1 M DTT, 0.1 M TRIS-Cl, pH 7.6) and lysed in heating shaker (95 °C/600 rpm) for 15 min.

The protein lysates were subjected to filter-aided sample preparation (FASP) using 10 kDa filters,^16^ and digested by trypsin (0.5 μg; sequencing grade; Promega). Peptides were then cleaned by liquid–liquid extraction (3 iterations) using water-saturated ethyl acetate.^17^ The cleaned FASP eluate was evaporated completely in a SpeedVac concentrator (Thermo Fisher Scientific) and dissolved in 15 μL of 50 mM ammonium bicarbonate with 0.1% SDS. Peptides were then cleaned with the SP2 protocol,^18^ and the eluates were evaporated in the SpeedVac concentrator. The resulting peptides were analyzed by LC-MS/MS.

The LC-MS/MS analyses of all peptides were performed using an UltiMate 3000 RSLCnano system (Thermo Fisher Scientific) connected to a timsTOF Pro spectrometer (Bruker). Prior to LC separation, tryptic digests were online concentrated and desalted using a trapping column (Acclaim PepMap 100 C18, dimensions 300 μm ID, 5 mm long, 5 μm particles; Thermo Fisher Scientific). After washing the trapping column with 0.1% formic acid, the peptides were eluted (flow rate -300 nL/min) from the trapping column onto an analytical column (Aurora C18, 75 μm ID, 250 mm long, 1.6 μm particles, heated to 50 °C; IonOpticks) by a linear gradient program (60 min, 3–42% of mobile phase B; mobile phase A: 0.1% formic acid in water; mobile phase B: 0.1% formic acid in 80% acetonitrile). Equilibration of both the trapping column and the analytical column was performed prior to sample injection to the sample loop. The analytical column was placed inside the Butterfly Heater (Phoenix s&t), and its emitter side was installed inside the CaptiveSpray ion source (Bruker) according to the manufacturer’s instructions.

MSn data were acquired in data independent acquisition (DIA) mode with a default method *m*/*z* range of 100–1700 and a 1/k0 range of 0.6–1.4 V × s × cm^–2^. The precursor range was defined from *m*/*z* 400–1000 with equal window sizes of 26 Th, using two steps in each PASEF scan and a cycle time of 100 ms locked to 100% duty cycle.

DiaPASEF data were processed in DIA-NN14 v1.8.1 ^19^ in library-free mode against a modified cRAP database (The common Repository of Adventitious Proteins; based on http://www.thegpm.org/crap; 112 sequences in total), the UniProtKB protein database for rabbit (21,176 protein sequences) and protein annotations of *T. pallidum* (see below*)*. During library preparation, no variable modifications were set, carbamidomethylation was set as a fixed modification, and the trypsin/P enzyme was used with one allowed missed cleavage. False discovery rate (FDR) control was set to 1%. MS1 and MS2 accuracies, as well as scan window parameters, were determined based on initial test searches (median value from all samples’ ascertained parameter values). Match-between-runs (MBR) was switched on.

The reported protein intensities were further processed using the software container environment (https://github.com/OmicsWorkflows). The processing workflow is available upon request. Briefly, it covered: a) removal of low-quality precursors and contaminant protein groups, b) outlying sample replicate removal, c) precursor intensity normalization by the loessF algorithm, d) filtered and normalized precursor intensities imputation (global 0.001 quantile), e) protein group MaxLFQ and DIA-TPA intensity calculation and their log_2_ transformation, and f) differential expression using the LIMMA statistical test.

The mass spectrometry proteomics data have been deposited to the ProteomeXchange Consortium via the PRIDE^20^ partner repository with the data set identifier PXD065022.

### Genome Annotation and ORF Prediction

Genome annotations were performed for seven *T. pallidum* strains using eight independent approaches to ensure comprehensive gene prediction coverage. These included seven automated pipelines: DFAST v1.3.6,^21^ PGAP v2024–07–18.build7555,^22^ Prodigal v2.6.3,^23^ Prokka v1.14.6,^24^ RAST v1.3.0,^25^ GeneMarkS-2 v1.25,^26^ and ORFfinder v0.4.3.^27^ In addition, the annotation downloaded from GenBank, representing the original manual annotation, was used for each strain.

All tools were used with default settings configured for prokaryotic genomes. In addition, ORFfinder was configured to predict open reading frames with a minimum length of 150 base pairs, with nested ORFs disabled. Prokka was performed with the genus-specific settings optimized for *Treponema* to improve annotation accuracy.

Annotations from all tools were integrated using Roary v3.13.0, which clustered protein-coding sequences into orthologous groups based on ≥ 90% amino acid identity. The Roary output was used to unify gene identifiers across annotations and to establish consistent gene names for downstream comparisons. To further refine ortholog assignments and resolve naming inconsistencies, all predicted protein sequences were aligned using BLASTp.^28^ High-confidence matches (e-value <1e^–10^) were used to assign common gene names across annotations and strains. The final reference proteome was evaluated in parallel during downstream peptide detection and statistical analysis.

### Descriptive Analysis

To evaluate detection consistency and proteome coverage, reference protein set for each *T. pallidum* strain was generated using all eight available annotations (see above). After removing nontreponemal peptides (an average of 9,592 rabbit peptides and 557 cRAP peptides per sample), the number of unique peptides was calculated for each strain-annotation combination. Peptides with more than one match were then omitted from further analysis (0.22% of treponemal peptides), and the number of detected proteins was determined. Additional summary statistics, such as peptide count per protein and the number of proteins supported by two or more peptides, were calculated to assess detection confidence. Detection rates were expressed as percentages relative to the number of annotated proteins.

To determine the extent of detected treponemal proteins, we compile an annotation of unique proteins using the Roary pipeline. These proteins were predicted across seven *T. pallidum* strains, integrating data from seven different annotation sets (excluding ORF prediction tool). Detected proteins were classified based on their presence across *T. pallidum* strains as either core (detected in all seven strains), shared (detected in two to six strains), or strain-specific (detected in only one strain).

### Quantitative Analysis

Quantitative proteomic comparisons were performed across seven *T. pallidum* strains. First, protein-coding gene annotations were generated for each strain using the RAST algorithm. These annotations were then merged into a unified reference set, which served as the basis for peptide and protein identification in the DIA-MS data sets. Protein group intensities were log_2_-transformed, and differential expression between strains was calculated using log_2_ fold-change (logFC) and adjusted p-values, employing the LIMMA statistical framework with multiple testing correction using Benjamini and Hochberg method.

To identify statistically significant protein expression differences, all pairwise comparisons were performed among the seven strains. Additionally, a grouped comparison was made between SS14-like strains (SS14, Philadelphia 1, Grady, Mexico A) and Nichols-like strains (DAL-1, Haiti B, Madras). Notably, strain-specific peptides encoded by TP0136 were excluded from all comparisons, and TP0136 itself was not evaluated.

### Pseudogene Annotation

In the *T. pallidum* genome, a number of pseudogenes have been identified.^13,29^ Pseudogenes analyzed in this study were defined as those that fulfilled the following criteria: (i) predicted gene length was longer than 415 bp to avoid misannotated open reading frames, (ii) nucleotide variants in the first 100 bp leading to frameshifts or premature stop codons were not considered due to the possible presence of a downstream start of the gene, and (iii) nucleotide variants in the last quarter of the gene were not considered due to the possible variable C-terminus length of the mature protein. Based on the inclusion criteria, three cases where both full-length genes and pseudogenes were present among the tested *T. pallidum* strains were identified: TP0127, TP0865, and TP0924.

## Results

### Proteome Analysis of Seven *In Vitro* Cultivated *T. pallidum* Strains

Seven *T. pallidum* strains (DAL-1, Madras, Haiti B, Mexico A, SS14, Grady, and Philadelphia 1) were analyzed in three biological replicates. The peptide detection during proteome analyses was performed using a manual annotation submitted to GenBank at the time of sequencing as well as annotations generated by various algorithms (DFAST, PGAP, Prodigal, Prokka, RAST, GeneMarkS) and an open reading frame predictor (ORFfinder) for the corresponding genomes. Treponemal cell counts in analyzed samples (ranging from 2.7 × 10^6^ to 20.0 × 10^6^, Table 2) generally corresponded to the number of detected unique peptides.

**Table 2:** Numbers of Detected Unique Peptides for Individual Replicates

	DFM^a^ counts of TPA cells			number of detected unique peptides^b^		
TPA strain	replicate 1	replicate 2	replicate 3	replicate 1	replicate 2	replicate 3
DAL-1	6.8 × 10^6^	17.0 × 10^6^	13.6 × 10^6^	3422	6867	4345
Madras	3.4 × 10^6^	10.2 × 10^6^	3.4 × 10^6^	3446	3492	3966
Haiti B	6.8 × 10^6^	17.0 × 10^6^	10.2 × 10^6^	928	6637	2901
Mexico A	20.0 × 10^6^	10.2 × 10^6^	4.8 × 10^6^	2893	4300	2320
SS14	17.0 × 10^6^	4.8 × 10^6^	3.4 × 10^6^	3986	3017	2880
Grady	4.1 × 10^6^	4.8 × 10^6^	2.7 × 10^6^	1488	2045	1856
Philadelphia 1	6.8 × 10^6^	10.2 × 10^6^	4.8 × 10^6^	3855	3216	6733

a DFM; dark-field microscopy.

b Mean value from all algorithms.

Through the combination of all annotations, 10,823 unique peptides of treponemal origin were detected across all analyzed *T. pallidum* strains. The number of detected peptides and their corresponding individual proteins varied across *T. pallidum* strains, depending on the annotation used. The RAST algorithm predicted the highest number of peptides for three strains (DAL-1, Mexico A, Philadelphia 1), followed by GeneMarkS (Haiti B, Grady) and Prodigal (Madras, SS14). At the protein level, RAST resulted in detection of the highest number of proteins for four strains (Madras, Haiti B, SS14, and Grady), followed by ORFfinder (Mexico A, Philadelphia 1) and Prodigal (DAL-1) (Table S1). Using the identified treponemal peptides/proteins from the RAST annotation (Table S1), the total number of detected peptides for individual strains ranged from 2101 to 7455, representing 605 (Grady) to 863 (Haiti B) detected proteins (Figure 1A and B). Altogether, the RAST detected 899 unique proteins out of 1,053 annotated proteins from all seven *T. pallidum* strains (85.4%). On average, 85.5% and 72.4% of identified proteins were detected by ≥ 2 and ≥ 3 peptides, respectively. The percentages of detected proteins with more than one peptide for individual strains are shown in Figure 1C. While RAST detected the highest number of individual proteins in most strains (4/7), GeneMarkS consistently provided the highest percentage coverage (up to 88.9%) of the annotated genomes when comparing detected proteins across different annotation algorithms (Figure 1D). Conversely, with the exception of ORFfinder, the RAST algorithm exhibited the lowest percentage of detected proteins. The Prokka algorithm showed a good balance between the absolute number of detected proteins and the percentage of detected annotated proteins (Table S1).

**Figure 1:**
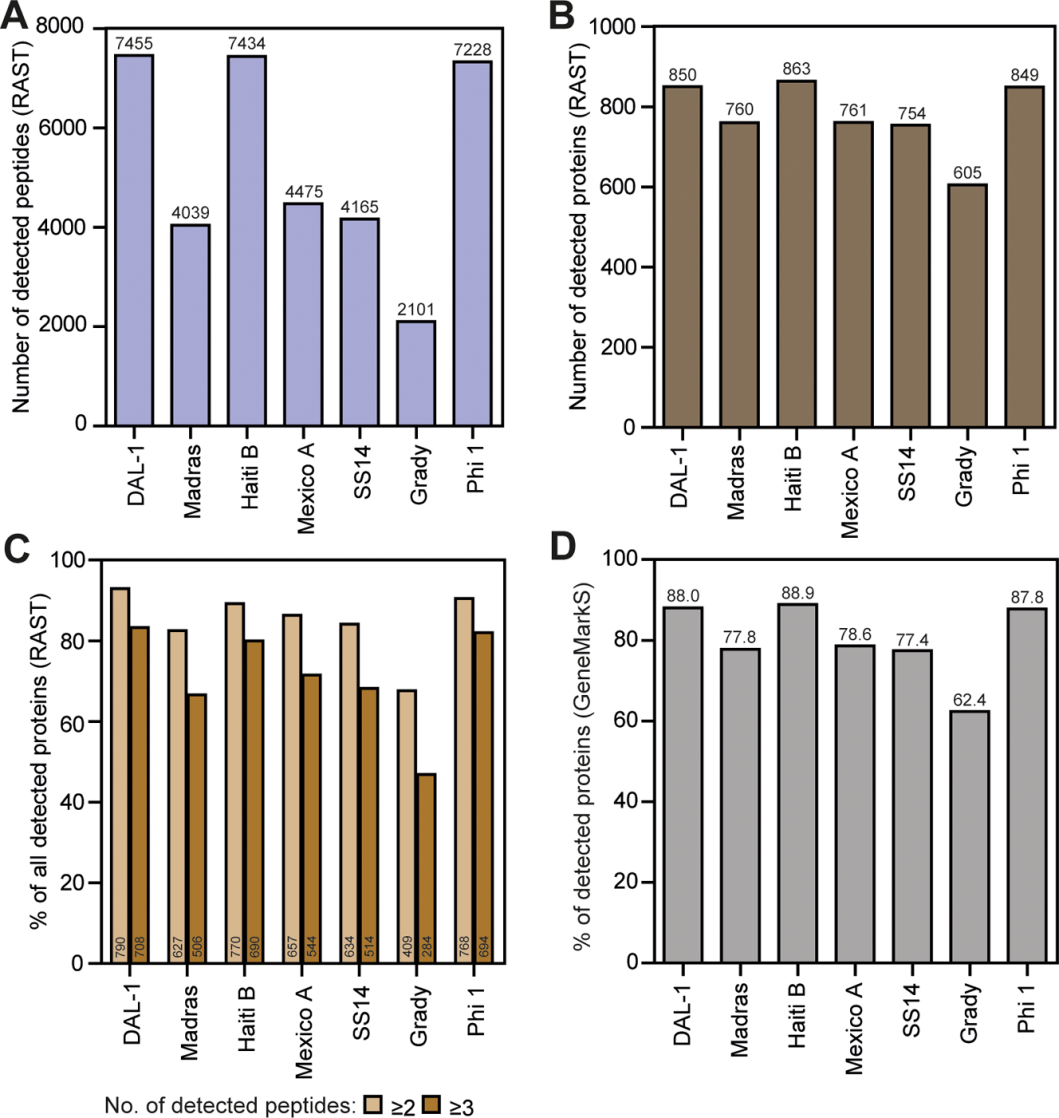
**Proteome analysis for individual** ***T. pallidum*** **strains.** The numbers of detected peptides (**A**) and proteins (**B**) are shown for seven *T. pallidum* strains (DAL-1, Madras, Haiti B, Mexico A, SS14, Grady, Philadelphia 1). Detection of most of the treponemal proteins was based on more than one identified peptide (**C**). The majority (up to 88.9%) of predicted proteins was identified in treponemal proteome (**D**). The numbers shown above or at the base of the columns represent the obtained values. The proteomic data obtained using the RAST algorithm are shown in panels A-C, while GeneMarkS data were used for panel D. The complete proteomic data from all tested algorithms are shown in Tables S1 and S2.

### Detection of Individual Proteins in the Analyzed Proteomes

To determine the extent of detected proteins, seven different annotations (excluding ORFfinder as a simple ORF prediction tool) of seven treponemal strains were combined together and only unique proteins were kept. Altogether, a set of 1,216 predicted proteins were used for database searching of the whole MS/MS data set and 911 proteins were identified (74.9%) (Figure 2A). Among them, 625 proteins were detected in all seven *T. pallidum* strains. Moreover, specific proteins were identified for strains DAL-1 (n = 4), Madras (n = 2), Haiti B (n = 14), and Philadelphia 1 (n = 7) (Figure 2A). The exclusion of strain Grady (which exhibited the lowest number of detected proteins, Figure 1D) expanded the treponemal core genome to 735 proteins (80.7% of 911 identified proteins; see Figure S1).

**Figure 2:**
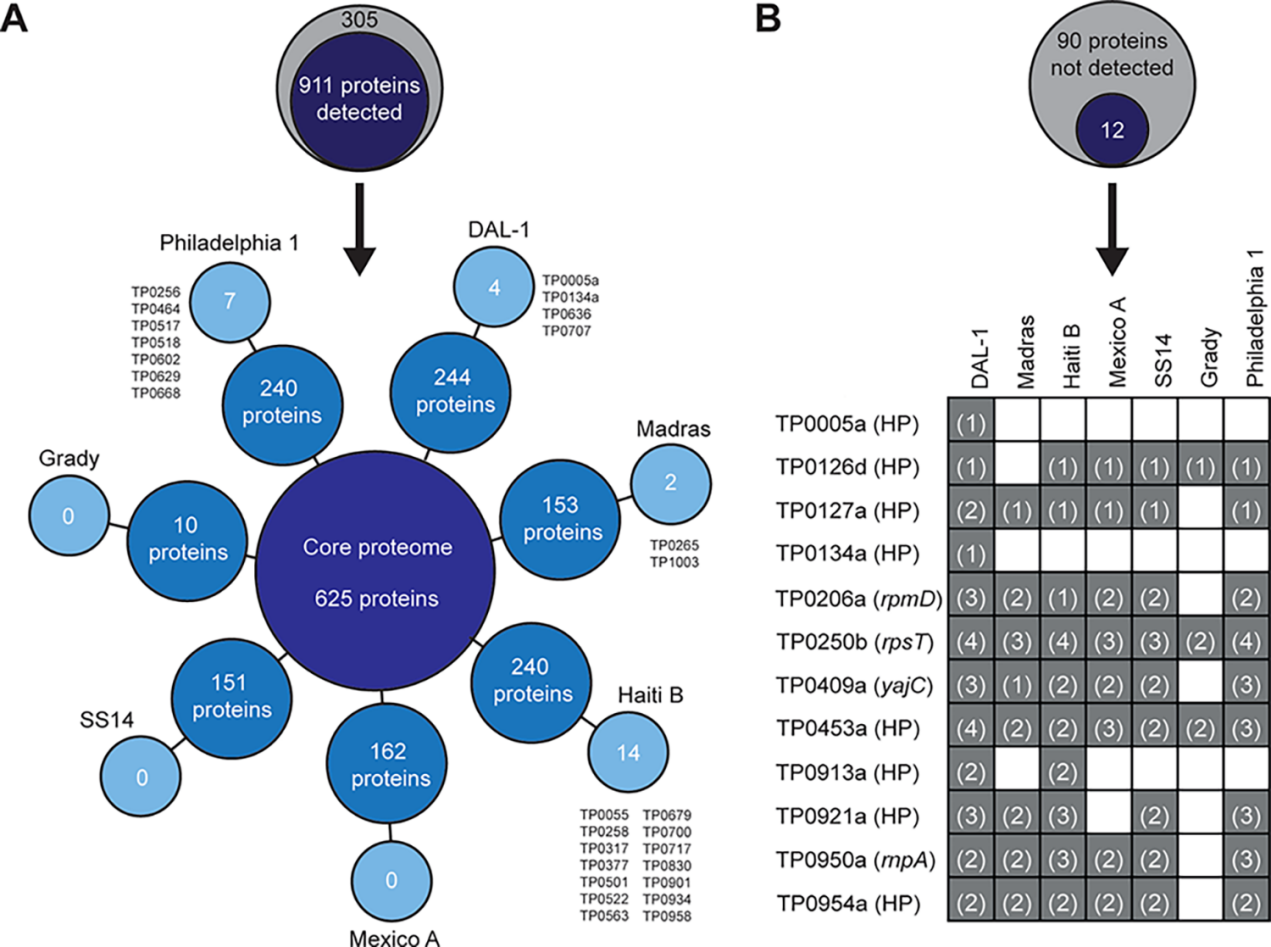
**An overview of detected proteins in the analyzed proteomes.** (**A**) An artificial annotation containing 1,216 predicted protein sequences was built based on all annotations used and all *T. pallidum* strains analyzed. Altogether, 911 proteins were detected in at least one treponemal strain. A scheme illustrates the distribution of detected *T. pallidum* proteins: the core proteome (inner circle) represents proteins identified among all seven strains; the middle circle represents "non-core" proteins also detected in other proteomes; and the outer circle represents proteins specifically found only in an individual *T. pallidum* strain. (**B**) Among 102 genes newly annotated in the DAL-1 (CP003115.1 ^30^) and Grady (CP035104.1 ^31^) genomes, peptides for 12 proteins were detected. Notably, ten of these proteins were detected across several *T. pallidum* strains. Number of detected unique peptides is shown in parentheses. HP, hypothetical protein.

In our previous studies,^30,31^ we annotated 102 new treponemal genes (i.e., genes that were named with a letter suffix) during DAL-1 and Grady genome sequencing. Among them, only 12 (11.8%) proteins were detected in the analyzed proteomes. Two proteins (TP0005a and TP0134a) were found only in DAL-1 strain, while the remaining ten proteins (TP0126d, TP0127a, TP0206a, TP0250b, TP0409a, TP0453a, TP0913a, TP0921a, TP0950a, TP0954a) were detected in several strains (Figure 2B).

### Detection of Pseudogene Products

In the genomes of analyzed *T. pallidum* strains, three predicted genes (TP0127, TP0865, TP0924) were found to be present as both full-length genes and pseudogenes (see inclusion criteria in Methods). Specifically, the full-length form of TP0127 and TP0924 was annotated in the Mexico A and DAL-1 genomes, respectively, while their pseudogene variants were found among other analyzed strains. For the TP0865 gene, the full-length form was found in all strains except for Haiti B. For TP0127, only two peptides were detected, both in the N-terminal region of its protein versions. In contrast, for TP0865 and TP0924, peptides were identified along the entire protein sequence. Surprisingly, peptides (two for TP0865 and one for TP0924) were detected even in the distal part of annotated pseudogenes, indicating that the mutations in these pseudogenes (TP0865: frameshift mutation; TP0924: mutation resulting in a stop codon) do not prevent translation of the downstream pseudogene sequence. The smaller proteins encoded by distal parts of the pseudogenes and detected in Haiti B and Madras were denoted as TP0865a and TP0924a, respectively (Figure 3).

**Figure 3:**
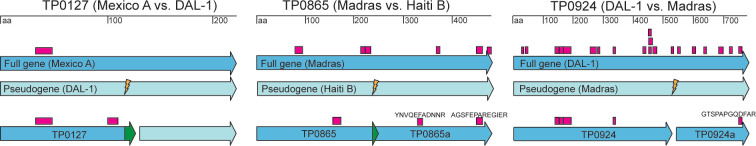
**An overview of detected peptides encoded by full-length genes and pseudogenes in loci TP0127, TP0865, and TP0924.** While only the proximal protein sequence was detected in TP0127, peptides (magenta) for both the proximal and distal parts of pseudogenes TP0865 and TP0924 were detected. The detected small distal proteins were denoted as TP0865a and TP0924a. For each analyzed locus, a single *T. pallidum* strain representing the full gene (blue) and a separate strain representing the corresponding pseudogene (light blue) were selected. Mutations resulting in pseudogene versions are indicated by a lightning bolt symbol. While mutations in TP0127 and TP0865 lead to a frameshift (green color), the mutation in TP0924 resulted in a stop codon.

### Quantitative Differences in Proteomes of Individual *T. pallidum* Strains

For comparative proteomic analysis, a merged RAST annotation of seven treponemal strains was used. Out of 16,177 proteomic comparisons between individual strains, 139 (0.86%) showed statistical significance (p-adj <0.05). Specifically, statistically significant quantitative differences were found for 51 proteins (see Table S3). Among them, 19 unique proteins showed an 8-fold or higher difference in abundance (log_2_ fold change >3) across various *T. pallidum* strains and are detailed in Figure 4.

**Figure 4:**
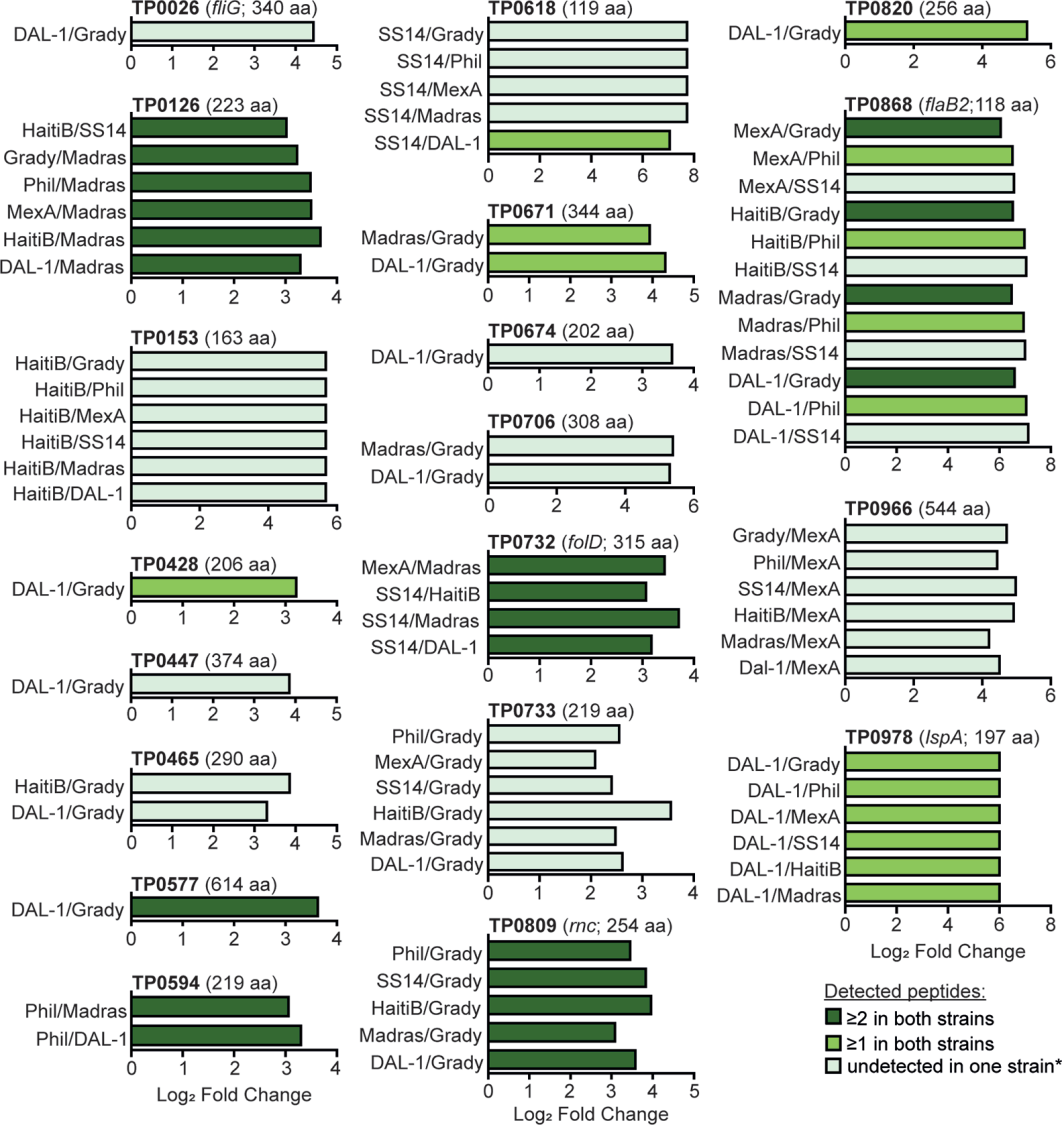
**Proteins with significant quantitative differences among** ***T. pallidum*** **strains**. Only statistically significant pairwise differences in protein abundance with a log_2_ fold change exceeding 3 are presented. *Undetected peptides were imputed with the global 0.001 quantile intensity. A complete list of 51 proteins with statistically significant changes (p-adj <0.05), represented by 139 statistically significant pairwise comparisons, is provided in Supplementary Table S3.

To determine if statistical differences were common across multiple comparisons between treponemal strains, we compared sequentially related SS14-like strains (SS14, Mexico A, Grady, and Philadelphia 1) and three Nichols-like strains (DAL-1, Madras, and Haiti B). Five proteins (products of TP0477, TP0487, TP0594, TP0597, and TP0732) showed significant quantitative differences between these two groups (p-adj <0.05; Figure 5). Among SS14-like strains, signal peptide protein (TP0594) and enzyme FolD (TP0732) showed higher levels. Conversely, 6-phosphogluconolactonase (TP0477), quinoprotein alcohol dehydrogenase (TP0487), and a hypothetical protein (TP0597) were more abundant in the Nichols-like strains. Interestingly, the identified proteins were mostly enzymes, including an enzyme of the pentose phosphate pathway, a redox enzyme, and an enzyme of one-carbon metabolism.

**Figure 5:**
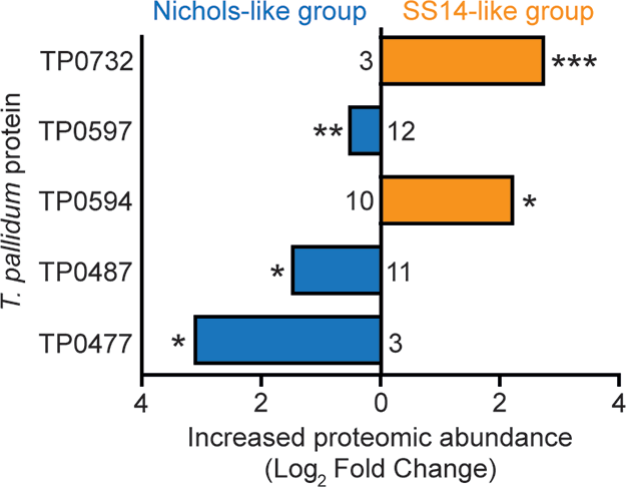
**Comparison of SS14-like and Nichols-like strains with respect to quantitative proteomic signal.** Five proteins (products of TP0477, TP0487, TP0594, TP0597, and TP0732) showed significant quantitative differences between these two groups. The numbers shown near the base of the columns represent the median number of detected peptides per strain. Note that the analysis was exclusively based on peptides with identical amino acid sequences across *T. pallidum* strains. The sequentially divergent protein TP0136, characterized by producing different peptides in different strains, was not evaluated. Significance was calculated using the LIMMA statistic with Benjamini-Hochberg correction (p-adj <0.05, ** p-adj <0.01, *** p-adj <0.001). For more details, see Table S4.

TP0732 (FolD) exhibited the highest statistical significance among the analyzed proteins, accompanied by a notable fold change. A sequence difference was found 36 nucleotides upstream of the TP0732 gene when comparing Nichols-like and SS-14-like strains (unlike with other identified loci; see Table S4). This finding suggests that potential variations in TP0732 gene expression levels may exist between these two groups of strains.

## Discussion

In this study, we characterized proteomes of seven *T. pallidum* strains grown under *in vitro* conditions. For each strain, we analyzed three biological replicates and when the replicates were combined, we detected 62.4–88.9% of all annotated proteins which is in accordance with previous proteome analyses.^5–7^ The differences observed in the protein coverage among different treponemal strains most likely reflect the amount of input material, sample purity with respect to contaminating eukaryotic cells and treponemal cell integrity. By combining seven annotation algorithms with the genomic sequences of every tested strain, we detected 911 individual treponemal proteins out of 1,216 predicted. This represents over 74.9% of all annotated proteins. However, the number of annotated proteins is rather overestimated due to the inclusion of manual annotations (e.g., DAL-1 has 1,059 annotated genes in the manual annotation deposited in the GenBank^30^) and also by the inclusion of strain-specific annotations (individual strains differ in individual annotated genes).

The highest number of detected annotated proteins in an individual *T. pallidum* strain was found for the Haiti B strain, annotated by GeneMarkS algorithm, which revealed 857 out of 964 annotated proteins (88.9%). This detection rate suggests that the *T. pallidum* genome is highly efficient in terms of protein expression, a situation that has already been predicted by previous transcriptome analyses showing that almost all annotated *T. pallidum* genes were transcribed.^32,33^ On the other hand, 36 annotated proteins with predicted functions and previously reported gene transcripts^32,33^ were not detected during LC-MS/MS proteome analysis of all *T. pallidum* strains in this study (see Table S5), suggesting low abundance of these proteins and/or inefficient detection of the corresponding peptides. In our study, the median size (143 aa) of the 305 predicted, but undetected, proteins was significantly smaller than the average size of all predicted proteins (304 aa).

While several previously published studies analyzed *T. pallidum* proteomes obtained from infected rabbits (including the Nichols and the DAL-1 strains),^3–6^ only two studies by Houston et al.^6,7^ analyzed treponemal proteomes from *in vitro* conditions for the Nichols and SS14 strains. Our study significantly expands the existing proteome data. We have increased available treponemal proteomes to a total of eight, regardless of their growth conditions. This collection now includes four Nichols-like strains (Nichols, DAL-1, Madras, Haiti B) and four SS14-like strains (SS14, Mexico A, Philadelphia 1, Grady). Furthermore, our work has enhanced the available proteomes of *T. pallidum* strains grown *in vitro.*

Our study evaluated seven distinct genome annotation algorithms (DFAST, PGAP, Prodigal, Prokka, RAST, GeneMarkS, GenBank annotation) and one ORF prediction tool (ORFinder) to assess their efficiency in identifying treponemal peptides and proteins (Table S1). Surprisingly, despite predicting over 9,700 ORFs per genome, whole-genome ORF prediction by ORFfinder did not yield the highest number of detected peptides and proteins across all *T. pallidum* strains analyzed. This outcome likely results from the nearly 10-fold greater number of predicted coding regions, which consequently leads to a considerably higher number of potentially encoded peptides. For optimal protein prediction, allowing the best identification success rate, Prodigal and GeneMarkS showed the best performance. This suggests that these algorithms, in comparison to others used in this study, predict better protein sizes, meaning they likely predict more accurate proximal alternative gene starts and more relevant protein-encoding DNA sequences. Therefore, for the analysis of *T. pallidum* genomes, Prodigal and GeneMarkS algorithms appear to be the most suitable predictors, as they most accurately predict coding regions while limiting the number of untranslated open reading frames. However, for the prediction of individual proteins, the best results were obtained with the RAST algorithm, which also predicted the highest number of genes per genome, with the exception of ORFfinder. The RAST algorithm should be used for the identification of the highest number of proteins, particularly in analyses where false gene predictions are not a primary concern.

The support for detected proteins was found to be robust, with 85.5% of detected proteins identified by at least two peptides and almost three-quarters of proteins identified by three or more peptides. This lowers the risk of falsely identified proteins. These findings are comparable with other *T. pallidum* proteome studies.^5–7^

The study by Houston et al.^6^ listed a set of 50 treponemal proteins that were not detected by any of the previous proteome studies analyzing Nichols, DAL-1, and SS14.^3–5^ Out of these 50 proteins, our study identified 13 (for more details see Table S6), theoretically leaving 37 predicted *T. pallidum* proteins that remain undetected by proteome analyses. However, the number of undetected proteins depends on the genome annotation algorithm(s) used, as demonstrated in our study. In any case, the minimalistic genome of *T. pallidum* appears to express most of its genetic information both under *in vivo* and *in vitro* growth conditions. On the other hand, the similar extent of the detected proteome in treponemes cultivated under *in vivo* and *in vitro* conditions does not preclude the existence of important quantitative proteome differences between them. However, answering this question would require a separate study simultaneously analyzing *in vivo* and *in vitro* proteomes from the same strain, using identical methods.

The seven analyzed *T. pallidum* strains differed in their set of three annotated pseudogenes (TP0127, TP0865, and TP0924). Intriguingly, for TP0865 and TP0924, we detected peptides corresponding to proteins encoded within the distal regions of these pseudogenes. Therefore, these pseudogenes could be expressed either via translational read-through of the mutation or by the original full-length protein being split into two separate proteins. A similar situation has already been described during genome analysis of *T. pallidum* ssp. *pertenue* strains,^34^ revealing that different genomes can harbor separate or fused gene versions. Our finding thus suggests that the protein functions normally provided by the full-length gene can instead be fulfilled by two proteins encoded by the pseudogene. The small proteins encoded by the distal parts of the pseudogenes were designated as TP0865a and TP0924a, and their presence should be considered in future studies.

Our study identified quantitative variations in several detected proteins across the analyzed *T. pallidum* strains. In total, 139 statistically significant differences were observed in individual strain-to-strain comparisons (Table S3). As transcriptomic data for these strains are unavailable, it remains uncertain whether these protein-level differences reflect variations in gene expression profiles among the treponemal strains. Nevertheless, previous research^14,35^ shows that individual strains differ in growth under *in vitro* conditions, and our observed proteomic differences might contribute these differences.

Nichols-like and SS14-like isolates differ in their genetic diversity within each group,^31,36^ the clinical characteristics of patients (e.g., syphilis stage),^37^ and the prevalence of macrolide resistance.^37^ These findings suggest important yet unrevealed genetic and physiological distinctions between them. Despite this, our proteomic comparison of three Nichols strains and four SS14-like strains identified only five significant differences: two proteins upregulated in SS14-like strains and three upregulated in Nichols-like strains. Proteins more abundant in the SS14-like strains included a signal peptide protein of unknown function and enzyme FolD, an enzyme of the one-carbon metabolism pathway involved in the biosynthesis of purine and thymidylate and other compounds. Proteins more abundant in the Nichols-like strains included one hypothetical protein and 6-phosphogluconolactonase, which is involved in the pentose phosphate pathway, important for the synthesis of nucleotides and nucleic acids. In addition, levels of quinoprotein alcohol dehydrogenase, catalyzing the oxidation of a range of substrates (including linear and aromatic primary and secondary alcohols, as well as aldehydes), were found elevated and may suggest differences in energy metabolism between both groups. The observed differences could result from sequence changes in the promoter/regulatory regions upstream of the corresponding genes (listed in Table S4); at least in the case of FolD, this explanation appears plausible.

However, several factors may explain the limited proteomic differences detected between these groups. A low number of replicates, a direct consequence of the challenges in *in vitro* treponeme cultivation, may have limited our ability to detect subtle differences. Alternatively, inherent proteome variability among the analyzed *T. pallidum* strains, or subtle proteomic changes that were below our detection limit, might be responsible for the previously described differences between the two clades. Future studies are needed to address this question. For instance, proteomic comparisons of the syphilis spirochete with other *T. pallidum* subspecies, which cause endemic treponematoses, could reveal proteomic differences related to their varied pathogenic and biological properties.

## Supplementary Material















## Data Availability

The mass spectrometry proteomics data have been deposited to the ProteomeXchange Consortium via the PRIDE^20^ partner repository with the data set identifier PXD065022.

## Notes

Figure S1. **Alternative overview of detected proteins (Excluding strain Grady, which had the lowest protein count).** Of 1,216 predicted protein sequences, 911 proteins were detected. The core proteome of six analyzed *T. pallidum* strains consists of 735 proteins (80.7%). A schematic representation is shown where the core proteome (inner circle) represents the proteins identified among all tested strains, the middle circle represents the proteins detected in two to five proteomes, and the outer circle represents proteins specifically found only in an individual *T. pallidum* strain (PDF)
Table S1. Numbers of *T. pallidum* peptides and proteins detected using 8 different annotations (XLSX)
Table S2. Numbers of detected individual treponemal proteins supported by the numbers of detected peptides (XLSX)
Table S3. Proteins with statistically significant quantitative differences (p-adj <0.05) in *T. pallidum* proteomes (XLSX)
Table S4. Quantitative proteomic differences between SS14-like (*n* = 4) and Nichols-like strains (*n* = 3) (XLSX)
Table S5. Undetected proteins with predicted functions and known gene expression levels (XLSX)
Table S6. Newly detected *T. pallidum* proteins (XLSX)
