# Supplementary material for: Proteome Analysis of Seven Treponema pallidum subsp. pallidum Strains Grown In Vitr o
Source: J Proteome Res. 2025 Oct 10;24(12):6091–100. doi: 10.1021/acs.jproteome.5c00624 (PMC12687308; doi:10.1021/acs.jproteome.5c00624)
Supplement: Supplementary file 1 [file pr5c00624_si_001.pdf]

Proteome analysis of seven *Treponema pallidum* subsp. *pallidum* strains grown *in vitro*

Juraj Bosák[1], Matěj Hrala[1], Klára Janečková[1], Kateřina Hanáková[2], Petra Pospíšilová[1], David Potěšil[2], Petr Andrla[1], Zbyněk Zdráhal[2], David Šmajs[1]*

[1]Department of Biology, Faculty of Medicine, Masaryk University, Brno, 625 00, Czech Republic

[2]National Centre for Biomolecular Research, Central European Institute of Technology and Faculty of Science, Masaryk University, Brno, 625 00, Czech Republic

*Corresponding author

E-mail: dsmajs@med.muni.cz

Short title: Proteome of *T. pallidum* strains

Supporting Information

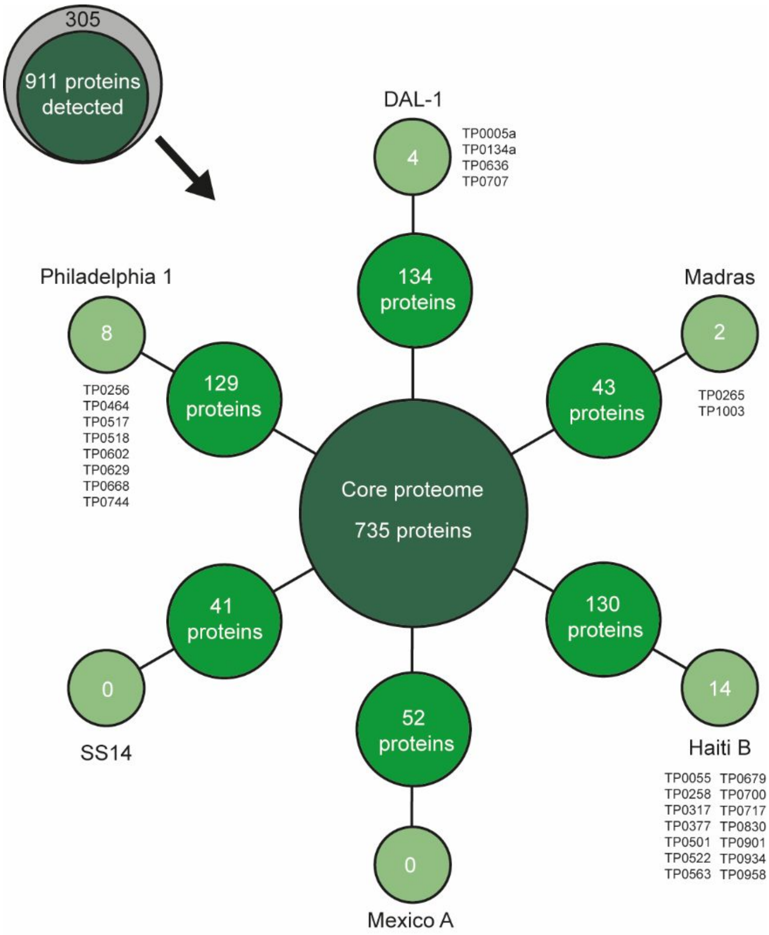

Figure S1. Alternative overview of detected proteins (Excluding strain Grady, which had the lowest protein count). Of 1,216 predicted protein sequences, 911 proteins were detected. The core proteome of six analyzed *T. pallidum* strains consists of 735 proteins (80.7%). A schematic representation is shown where the core proteome (inner circle) represents the proteins identified among all tested strains, the middle circle represents the proteins detected in two to five proteomes, and the outer circle represents proteins specifically found only in an individual *T. pallidum* strain.

Supporting Information

Table S1: Numbers of *T. pallidum* peptides and proteins detected using 8 different annotations. (XLSX)

Table S2. Numbers of detected individual treponemal proteins supported by the numbers of detected peptides. (XLSX)

Table S3. Proteins with statistically significant quantitative differences (p-adj<0.05) in *T. pallidum* proteomes. (XLSX)

Table S4. Quantitative proteomic differences between SS14-like (n = 4) and Nichols-like strains (n = 3). (XLSX)

Table S5. Undetected proteins with predicted functions and known gene expression levels. (XLSX)

Table S6. Newly detected *T. pallidum* proteins. (XLSX)
